# An economical and safe procedure to synthesize 2-hydroxy-4-pentynoic acid: A precursor towards ‘clickable’ biodegradable polylactide

**DOI:** 10.3762/bjoc.10.139

**Published:** 2014-06-17

**Authors:** Quanxuan Zhang, Hong Ren, Gregory L Baker

**Affiliations:** 1 Department of Chemistry, Michigan State University, East Lansing, Michigan 48824, USA; 2 Department of Chemistry and Chemical Biology, Harvard University, Cambridge, Massachusetts 02138, USA; 3 Athinoula A. Martinos Center for Biomedical Imaging, Department of Radiology, Massachusetts General Hospital, Harvard Medical School, Charlestown, Massachusetts 02129, USA

**Keywords:** alkylation, ‘click’ chemistry, ‘clickable’ polylactide, decomposition, diethyl 2-acetamidomalonate, 2-hydroxy-4-pentynoic acid, one-pot, optimization, propargyl bromide, propargyl tosylate, safe and economical

## Abstract

2-Hydroxy-4-pentynoic acid (**1**) is a key intermediate towards ‘clickable’ polylactide which allows for efficient introduction of a broad range of pendant functional groups onto polymers from a single monomer via convenient ‘click’ chemistry with organic azides. The incorporation of various pendant functional groups could effectively tailor the physicochemical properties of polylactide. The reported synthesis of **1** started from propargyl bromide and ethyl glyoxylate. However, both of starting materials are expensive and unstable; especially, propargyl bromide is shock-sensitive and subjected to thermal explosive decomposition, which makes the preparation of **1** impractical with high cost and high risk of explosion. Herein, we report a simple, economical and safe synthetic route to prepare **1** using cheap and commercially available diethyl 2-acetamidomalonate (**4**) and propargyl alcohol. The desired product **1** was obtained via alkylation of malonate **4** with propargyl tosylate followed by a one-pot four-step sequence of hydrolysis, decarboxylation, diazotization and hydroxylation of propargylic malonate **5** without work-up of any intermediate.

## Introduction

Aliphatic polyesters, in particular polylactide (PLA), are now widely used for biomedical applications, such as surgery sutures [1], implants for bone fixation [2], drug delivery vehicles [3–4] and tissue engineering scaffolds [5] because of their excellent biocompatibility, biodegradability and mechanical properties. However, due to low solubility of PLA in water, applications of PLA in an aqueous environment have been greatly restricted. In addition, the lack of functional group diversity in the backbones and side chains of PLA makes further modification difficult. Therefore, the introduction of a variety of functional groups to PLA is highly desirable to modulate the physicochemical properties of the resulting polymers, such as hydrophilicity.

Recently, numerous α,ω-chain end functionalized PLAs have been reported through ring-opening polymerization of lactide by judicious choice of initiating alcohols and further post-polymerization modification of ω-chain end hydroxy groups [6–8]. Moreover, the appending functionalities along PLA backbones provide great opportunities for altering physical and/or chemical properties of PLAs. PLA with pendent functional groups can be achieved by ring-opening polymerization of functional lactide monomers, post-polymerization modification, or a combination of these two approaches. The appending hydroxy [9], carboxyl [10], poly(ethylene glycol) (PEG) [11–14], allyl [15], azido [16] and acetylene [17] functionalities on PLA backbones have been reported and offered great opportunities for covalent post-polymerization modification of PLAs.

**Scheme 1:**
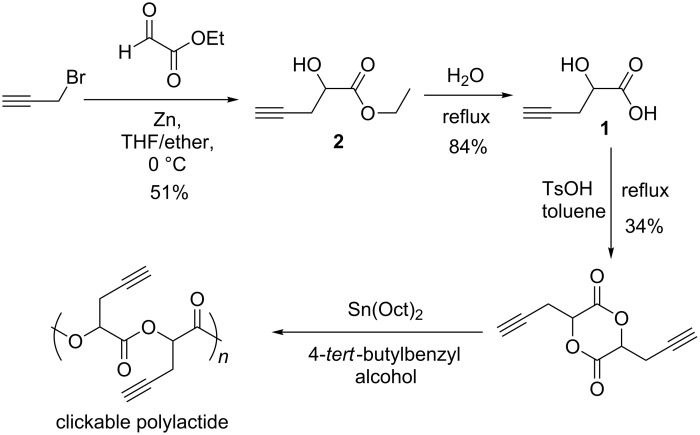
Synthesis of 2-hydroxy-4-pentynoic acid (**1**).

Among various functional groups, acetylene functionalized PLA has attracted much attention [17–19]. By having a single functional lactide monomer, it allows for facile placement of a broad range of pendant functional groups onto polymers via convenient ‘click’ reaction with organic azido molecules without PLA backbone degradation [17]. ‘Click’ chemistry [20], which is copper(I)-catalyzed 1,3-dipolar cycloaddition of azides and alkynes, has been developed and utilized in recent years as a powerful synthetic strategy because of its high selectivity and excellent tolerance to different functional groups and reaction conditions. Our group has prepared the first ‘clickable’ PLA using 2-hydroxy-4-pentynoic acid (**1**) as a precursor (Scheme 1) and studied ‘click’ modification of acetylene functionalized PLA [17]. One new family of water-soluble and temperature responsive biodegradable PLA material with tunable lower critical solution temperature (LCST) in a range from 25 to 65 °C was obtained after ‘click’ grafting with a mixture of alkyl and PEG azides. Starting from the same precursor **1** [18], Yu and coworkers have prepared a novel graft polymer-drug conjugate (GPDC) with potent cancer inhibitor paclitaxel linked to PLA backbones. The GPC results indicated that GPDC released paclitaxel moieties from polymer complexes in phosphate buffer solutions under physiological pH of 5.5 and 7.4, suggesting its potential clinical application. More recently, Coumes and coworkers have prepared acetylene functionalized PLAs from precursor **1** and ‘click’ modification of the resulting PLAs with PEG azides provided amphiphilic graft copolymers which form nanorod aggregates in water [19].

The results discussed above indicate the great power to tune physicochemical properties of PLA by ‘click’ modification of acetylene functionalized PLA. Precursor **1** is the key intermediate in the preparation of these ‘clickable’ PLAs. It was synthesized in 40% overall yield via hydrolysis of ester **2** which was generated by Reformatsky-type reaction of propargyl bromide with fresh distilled ethyl glyoxylate in the presence of activated zinc (Scheme 1) [17]. However, the synthesis of precursor **1** used propargyl bromide and ethyl glyoxylate as starting materials, both of which are expensive and unstable. These starting materials must be stabilized by toluene and purified before reaction. Flash column chromatography is also required in the process. Besides its high cost, propargyl bromide is shock-sensitive and subjected to thermal explosive decomposition [21–23], which makes the preparation of **1** via this method impractical with high risk of explosion.

**Scheme 2:**
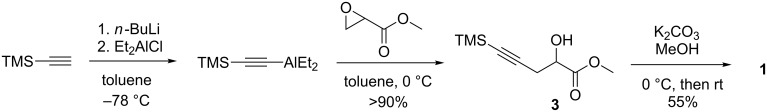
Synthesis of **1** via epoxide ring opening with organoalane.

Thus, efforts were taken to develop a safe and practical synthetic route for precursor **1**. Our first successful effort in the synthesis of **1** gave a good overall yield (50%) after removal of the trimethylsilyl protecting group and hydrolysis of ester **3**, generated from diethylaluminum(trimethylsilyl)acetylide and methyl glycidate (Scheme 2) [24]. Although it does not involve explosive propargyl bromide, this method requires an excess (2 equivalents) of expensive trimethylsilylacetylene [25–28] and highly flammable chemicals (butyllithium and diethylaluminum chloride) which makes this route even more costly and less attractive. Therefore, in order to provide sufficient **1** for preparing ‘clickable’ PLA and expand its application scope via ‘click’ modification in biomedical fields, an economical and safe procedure to synthesize **1** with good yield is highly desirable using simple and cheap starting materials.

**Scheme 3:**
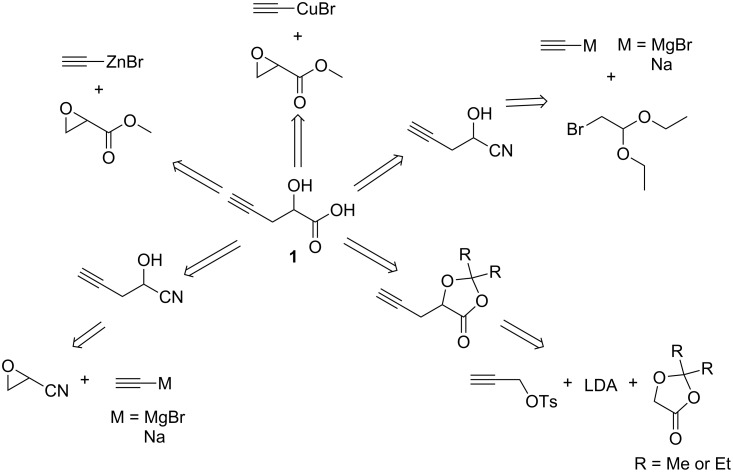
Attempted synthetic routes for compound **1**.

Several other promising synthetic routes to precursor **1** were also proposed and examined in our group in order to find a safe and affordable procedure without using propargyl bromide. However, all attempted synthetic routes to **1** indicated in Scheme 3 were determined not to be feasible probably due to the interruption of the ethynyl proton in the presence of organometallic reagents [29].

As discussed above, there are two important factors that should be taken into account in order to achieve a safe and economical route resulting in precursor **1**: 1) a non-explosive and cheap source of acetylene building moiety; and 2) basic reaction conditions involving a low p*K*a (<24) to avoid the interruption of the ethynyl proton (p*K*a ~24) while introducing the acetylene moiety. Propargyl tosylate, prepared from propargyl alcohol and tosyl chloride, is a safe analogue of propargyl bromide with low cost. Malonate has a low p*K*a of ~13 and can be easily deprotonated and alkylated by mild bases such as sodium/potassium alkoxides without deprotonating a terminal alkyne. Conveniently, both malonate and propargyl alcohol are commercially available in bulk quantities. Herein, we report one economical and safe procedure using diethyl 2-acetamidomalonate (**4**) and propargyl alcohol as starting materials to synthesize **1**. The synthesis was completed via alkylation of malonate **4** with propargyl tosylate followed by a one-pot four-step sequence of hydrolysis, decarboxylation, diazotization and hydroxylation of propargylic malonate **5** without work-up of any intermediates.

## Results and Discussion

**Scheme 4:**
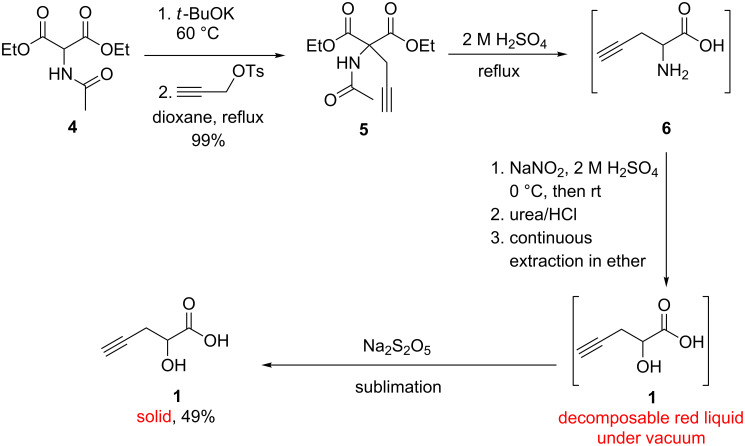
Synthesis of 2-hydroxy-4-pentynoic acid (**1**) from diethyl 2-acetamidomalonate.

The synthesis of the desired precursor **1** was straightforward and outlined in Scheme 4. Propargyl alcohol, a cheap source of an acetylene moiety, was tosylated with tosyl chloride following a modified procedure [30] to provide propargyl tosylate in high yield as a dark liquid after drying under vacuum. Diethyl 2-acetamidomalonate (**4**), deprotonated by potassium *tert*-butoxide, was alkylated in dioxane by a conventional *C-*alkylation method [31–34] with propargyl tosylate to give propargylic derivative **5**. The alkylation step was optimized and it can be conducted with an enolate concentration of **4** up to 0.25 M in dioxane (Table 1). The attempt with higher enolate concentration was not smooth due to the increased viscosity of the reaction mixture and thus low efficiency of stirring, even with a mechanical stirrer, which resulted in a messy mixture of product observed from the ^1^H NMR spectrum of the crude reaction mixture. The *C*-alkylation of **4** in a more common solvent, THF, was found to be not suitable due to the low conversion with even a much longer reaction time (entry 3, Table 1) [31]. Under the conditions indicated in Scheme 4 and Table 1, propargylic derivative **5** was obtained in quantitative yield and with high purity after optimization, and it is not necessary to further purify **5**.

**Table 1:** Optimization of alkylation of **4**.^a^

Entry	**4**/mmol	Solvent	Solvent volume/mL	Concentration of **4**/M	*t*/h	Conversion^b^	Yield

1	47.5	dioxane	475	0.1	20	98%	96.0%
2	95	dioxane	375	0.25	24	99%	99.0%
3	47.5	THF	475	0.1	120	55%	–^c^

^a^Potassium *tert*-butoxide was dissolved in solvent (half volume of total solvent) and transferred to a solution of **4** via cannula, then propargyl tosylate was added dropwise. ^b^Estimated by ^1^H NMR. ^c^Product was not isolated.

Reflux of **5** in a 2 M aqueous solution of H_2_SO_4_ enabled the one-pot synthesis of intermediate **6** via sequential hydrolysis of amide and esters, and decarboxylation of the resulting malonic acid. The resulting intermediate **6** was not isolated and used directly for the subsequent reaction. Notably, basic treatment of **5** as discussed in literature [31,33] would not allow conversion of **5** to **6** in a convenient one-pot manner, since more harsh basic conditions are required for carrying out amide hydrolysis of **5**. Because the following diazotization of **6** requires strong acidic conditions, the proper choice of acidic hydrolysis and decarboxylation of **5** in H_2_SO_4_ thus also further facilitated the one-pot conversion of intermediate **6** to **1** via diazotization and hydroxylation without additional work-up or isolation of **6** [35–40]. In addition, proper choice of H_2_SO_4_ instead of HCl [41–42] for one-pot conversion of **5** to **6** could avoid the formation of the possible side product 2-chloro-4-pentynoic acid [43–44], if chloride anions are present in the solution during the synthesis of **1** from **6** via diazotization and hydroxylation.

However, 2-hydroxy-4-pentynoic acid (**1**) rapidly decomposes while drying under high vacuum after continuous extraction from aqueous solution with diethyl ether (Table 2). This was not expected since acid **1** prepared by other methods did not decompose upon isolation (Scheme 2) [17]. The possible reason for this decomposition is that there might be certain oxidants present in ether phase after continuous extraction (for example, HNO_2_ and HNO_3_, verified by a rapid color change of KI-starch test paper), which destroy product **1** during concentration and drying under vacuum. And neither addition of urea (reacting with HNO_2_ to release N_2_, CO_2_ and H_2_O) nor passing of crude product **1** through a short silica gel column can stop its decomposition. So three reductants were examined which presumably would remove any residual oxidants. Product **1** was not detected in ether filtrate after addition of solid Na_2_SO_3_ to an ether solution of **1** and filtration to remove Na_2_SO_3_ solid. However, acid **1** can be recovered in lower yield after extracting the acidified aqueous solution of Na_2_SO_3_ with ether (Table 2). Apparently, acid **1** precipitated as its sodium salt from ether via an acid–base reaction with Na_2_SO_3_ (p*K*a of **1**: ~3.8 [45]; p*K*a of H_2_SO_3_: 1.9, 7.2). When reductant Na_2_S_2_O_3_ was added to an ether solution of acid **1**, a yellow solid mixture containing both acid **1** and sulfur was obtained after removal of ether under vacuum (Table 2). It is known that sodium thiosulfate decomposes to give sulfur as a solid product under acidic conditions [46]. Dissolution of this solid mixture in water, filtration to remove sulfur and subsequent removal of water in vacuo gave pure acid product **1** in 30% yield. These results indicate that it is not convenient to use solid Na_2_SO_3_ or Na_2_S_2_O_3_ to prevent the decomposition of **1**, since the addition of either salt complicated the following work-up and purification procedure. However, intact product **1** was readily obtained as a slight yellow solid in 49% yield by adding Na_2_S_2_O_5_ solid to an ether solution of acid **1**, followed by simple filtration, concentration and sublimation under vacuum (Table 2). The spectroscopic data of **1** were in good agreement with both authentic samples of **1** previously prepared in our lab and literature reports [17–19]. This indicates that solid Na_2_S_2_O_5_ removes possible oxidants and simply enables the formation of undecomposed solid product **1** after drying in vacuo. Despite the moderate yield, this procedure represents a simple, economical and safe approach to synthesize precursor **1**.

**Table 2:** 2-Hydroxy-4-pentynoic acid (**1**) after treatment with different reductants.

	Reductant^a^			
	
	None	Na_2_SO_3_	Na_2_S_2_O_3_	Na_2_S_2_O_5_

Compounds in ether phase^b^	**1**	No **1**	Sulfur + **1**	**1**
Appearance of dry **1**^c^	Decomposition black	Light yellow solid^d^	Yellow solid	Light yellow solid

^a^Solid reductants were added into ether extract containing **1** and the resulting mixture was stirred. ^b^Compounds in ether extract after treatment with reductants. ^c^Product **1** was obtained upon removal of ether after treatment with reductants and followed by filtration. ^d^Product **1** was recovered with low yield after extracting the acidified aqueous solution of solid Na_2_SO_3_ with ether.

## Conclusion

One simple, economical and safe procedure to prepare 2-hydroxy-4-pentynoic acid (**1**) was developed and optimized using cheap and commercially available starting materials – diethyl 2-acetamidomalonate (**4**) and propargyl alcohol. *C*-Alkylation of malonate **4** with propargyl tosylate in dioxane afforded propargyl derivative **5** quantitatively. The proper choice of acidic hydrolysis and decarboxylation of **5** in 2 M H_2_SO_4_ enabled the synthesis of precursor **1** via a one-pot four-step sequence of hydrolysis, decarboxylation, diazotization and hydroxylation of propargylic malonate **5** without work-up of any intermediates. Solid Na_2_S_2_O_5_ was effective to remove any possible oxidants (like HNO_2_ and HNO_3_) to prevent decomposition of product **1** while drying under vacuum. Overall, this procedure represents a practical and economical approach to conveniently synthesize precursor **1** for preparation of ‘clickable’ polylactide biomaterials.

## Experimental

### General methods

All reactions, if not stated otherwise, were performed in oven-dried glassware under nitrogen atmosphere. THF and dioxane were dried over sodium/benzophenone and distilled before use. All other reagents and solvents were ACS grade and used as received unless specified. Melting points were measured on an Electrothermo^®^ melting point apparatus. ^1^H NMR and ^13^C NMR were recorded on a VXR-500 MHz instrument in CDCl_3_ unless otherwise noted. CDCl_3_ was used as the internal standard for both ^1^H NMR (δ = 7.24) and ^13^C NMR (δ = 77.0).

#### Synthesis of propargyl tosylate

A 2 L round bottom flask equipped with a mechanical stirrer was charged with 58 mL (1.0 mol) of propargyl alcohol, 250 g (1.30 mol) of tosyl chloride and 1000 mL of diethyl ether under nitrogen. The resulting reaction mixture was cooled in an ice bath, and NaOH pellets (200 g, 5.00 mol) were added to the solution in 6 portions at 0 °C under vigorous stirring. The resulting mixture was continually stirred overnight at room temperature. The suspension was poured into cold water and the resulting aqueous layer was extracted with ether (2 × 250 mL). The ether layer was combined, dried over anhydrous Na_2_SO_4_ and concentrated. Pure propargyl tosylate was obtained as a dark liquid [30,47] in 84.0% yield (185 g) by drying under high vacuum (Caution: gloves are required when handling propargyl tosylate). ^1^H NMR (500 MHz, CDCl_3_) δ 7.76 (d, *J* = 8 Hz, 2H), 7.30 (d, *J* = 8.5 Hz, 2H), 4.65 (d, *J* = 2.5 Hz, 2H), 2. 49 (t, *J* = 2.5 Hz, 1H), 2.47 (s, 3H); ^13^C NMR (125 MHz, CDCl_3_) δ 145.3, 133.1, 130.0, 128.2, 77.4, 75.4, 57.44, 21.8.

#### Synthesis of diethyl α-propargyl-α-acetamidomalonate (**5**)

To a solution of 105 g (0.483 mol) of diethyl 2-acetamidomalonate (**4**) in 1.35 L of dioxane was added a slurry of 61 g (0.54 mol) of potassium *tert*-butoxide in 550 mL of dioxane dropwise via cannula over 2 h while stirring with a mechanical stirrer at room temperature. The resulting suspension was heated to 50 °C and stirred for another 2 h. A solution of propargyl tosylate (83 mL, 0.49 mol) in 150 mL of dioxane was added dropwise at 50 °C over 1 h and the resulting mixture was brought to reflux overnight. The reaction mixture was cooled to room temperature and filtered to remove the solid. The filtrate was concentrated on rotavap and the crude product was dissolved in 1 L of dichloromethane. The organic layer was washed with water (2 × 500 mL), decolorized with activated charcoal and dried over anhydrous Na_2_SO_4_. The desired product diethyl α-propargyl-α-acetamidomalonate (**5**) was obtained as a light yellow solid [31] in 99.0% yield (122 g) by solvent evaporation and drying under vacuum. Mp 85–88 °C; ^1^H NMR (500 MHz, CDCl_3_) δ 6.91 (s, 1H), 4.24 (dd, *J* = 3, 7.5 Hz, 4H), 3.26 (d, *J* = 2.5 Hz, 2H), 2.04 (s, 3H), 1.94 (t, *J* = 2.5 Hz, 1H), 1.24 (t, *J* = 7.5 Hz, 6H); ^13^C NMR (125 MHz, CDCl_3_) δ 169.6, 166.8, 78.7, 71.9, 65.5, 63.3, 24.1, 23.3, 14.2.

#### Synthesis of 2-hydroxy-4-pentynoic acid (**1**)

A 3 L round bottom flask was charged with 110 g (0.431 mol) of **5** and 1.2 L of 2 M H_2_SO_4_ and the resulting mixture was heated to reflux until full conversion of **5** to 2-amino-4-pentynoic acid (**6**, ~36 h). The reaction mixture was used directly without further isolation. A solution of NaNO_2_ (5 equiv, 40%) in water was added dropwise to the aqueous solution of amino acid **6** at 0 °C followed by the addition of another 3 equiv of NaNO_2_ in water (40%). The resulting mixture was stirred for 40 h at room temperature, and urea in diluted HCl (1 M) was added dropwise until the mixture did not make potassium iodide starch test paper blue or purple. The resulting product **1** was extracted with ether using a continuous extraction apparatus. Solid Na_2_S_2_O_5_ was added to ether layer and the resulting mixture was stirred until it did not turn potassium iodide starch test paper to purple. After removal of Na_2_S_2_O_5_ via filtration, the ether layer was concentrated, dried and sublimated under vacuum to give 24.1 g of acid **1** in 49.0% yield [17]. ^1^H NMR (500 MHz, CDCl_3_) δ 4.35 (t, *J =* 4.5 Hz, 1H), 2.68 (m, 2H), 2.08 (t, *J* = 2.5 Hz, 1H); ^13^C NMR (125 MHz, CDCl_3_) δ 177.5, 77.7, 71.9, 68.5, 24.4.
